# Co-release of noradrenaline and dopamine in the cerebral cortex elicited by single train and repeated train stimulation of the locus coeruleus

**DOI:** 10.1186/1471-2202-6-31

**Published:** 2005-05-02

**Authors:** Paola Devoto, Giovanna Flore, Pierluigi Saba, Mauro Fà, Gian Luigi Gessa

**Affiliations:** 1 Department of Neuroscience "B.B. Brodie" University of Cagliari, Italy; 2 Institute of Neuroscience, Consiglio Nazionale delle Ricerche, Section of Cagliari, Cagliari, Italy; 3 Centre of Excellence "Neurobiology of Addiction", University of Cagliari, Cagliari, Italy

## Abstract

**Background:**

Previous studies by our group suggest that extracellular dopamine (DA) and noradrenaline (NA) may be co-released from noradrenergic nerve terminals in the cerebral cortex. We recently demonstrated that the concomitant release of DA and NA could be elicited in the cerebral cortex by electrical stimulation of the locus coeruleus (LC). This study analyses the effect of both single train and repeated electrical stimulation of LC on NA and DA release in the medial prefrontal cortex (mPFC), occipital cortex (Occ), and caudate nucleus. To rule out possible stressful effects of electrical stimulation, experiments were performed on chloral hydrate anaesthetised rats.

**Results:**

Twenty min electrical stimulation of the LC, with burst type pattern of pulses, increased NA and DA both in the mPFC and in the Occ. NA in both cortices and DA in the mPFC returned to baseline within 20 min after the end of the stimulation period, while DA in the Occ reached a maximum increase during 20 min post-stimulation and remained higher than baseline values at 220 min post-stimulation. Local perfusion with tetrodotoxin (TTX, 10 μM) markedly reduced baseline NA and DA in the mPFC and Occ and totally suppressed the effect of electrical stimulation in both areas.

A sequence of five 20 min stimulations at 20 min intervals were delivered to the LC. Each stimulus increased NA to the same extent and duration as the first stimulus, whereas DA remained elevated at the time next stimulus was delivered, so that baseline DA progressively increased in the mPFC and Occ to reach about 130 and 200% the initial level, respectively.

In the presence of the NA transport (NAT) blocker desipramine (DMI, 100 μM), multiple LC stimulation still increased extracellular NA and DA levels.

Electrical stimulation of the LC increased NA levels in the homolateral caudate nucleus, but failed to modify DA level.

**Conclusion:**

The results confirm and extend that LC stimulation induces a concomitant release of DA and NA in the mPFC and Occ.

The different time-course of LC-induced elevation of DA and NA suggests that their co-release may be differentially controlled.

## Background

Noradrenergic neurons in the locus coeruleus (LC) project homogeneously throughout the cerebral cortex, while dopaminergic afferents are mainly confined to discrete areas of the cortex, such as the medial prefrontal cortex (mPFC), anterior cingulate, rhinal and entorhinal cortices [1,2]. However, even in cortical areas where dopaminergic and noradrenergic innervations converge, such as the mPFC, tissue noradrenaline (NA) concentrations exceed those of dopamine (DA). Furthermore, in the cerebral cortex a consistent mismatch exists between DA receptor distribution and dopaminergic innervation, DA receptors being widely expressed in non-innervated areas [3-7].

Previous evidence from our laboratory has suggested the possibility that DA in the cerebral cortex may be released other than from dopaminergic terminals also from noradrenergic ones, where DA acts both as NA precursor and co-transmitter [8-12]. This hypothesis is supported by the fact that extracellular DA concentrations in cortical areas scarcely innervated by the meso-cortical dopaminergic pathway, such as the occipital (Occ) and parietal cortices, were found to be only slightly lower than in the mPFC, an area with massive dopaminergic afferents [8].

Moreover, extracellular DA in different cortices was found to be modified by drugs acting on noradrenergic transmission but not, or only modestly, by drugs modifying dopaminergic activity [9-11]. Thus, consistent with its ability to inhibit noradrenergic activity, the α_2_-adrenoceptor agonist clonidine produced a concomitant reduction in extracellular DA and NA both in the mPFC and Occ, whereas the α_2_-adrenoceptor antagonists idazoxan and RS 79948 produced a concomitant increase in extracellular NA and DA in both cortices [10,11]. Moreover, local infusion into the LC of carbachol, kainic acid or N-methyl aspartic acid increased both NA and DA in the mPFC, in accord with their ability to activate noradrenergic neurons [13].

In a previous study [12] we demonstrated that extracellular NA and DA in the cerebral cortex are increased by electrical stimulation of the LC in a frequency dependent manner. However, the higher frequency patterns tested elicited a robust behavioural activation, and even at low frequencies animals displayed a state of alert with fine muscle contractions and whisker vibrations. As stress has been demonstrated to increase both NA and DA in the cerebral cortex [14-16], to rule out the possibility that the effect of LC stimulation might be influenced by stress, we performed the present study in anaesthetized rats. Moreover, we studied whether the release of DA and NA might be differentiated by applying single train or repeated train stimulations.

## Results

**Figure 1:**
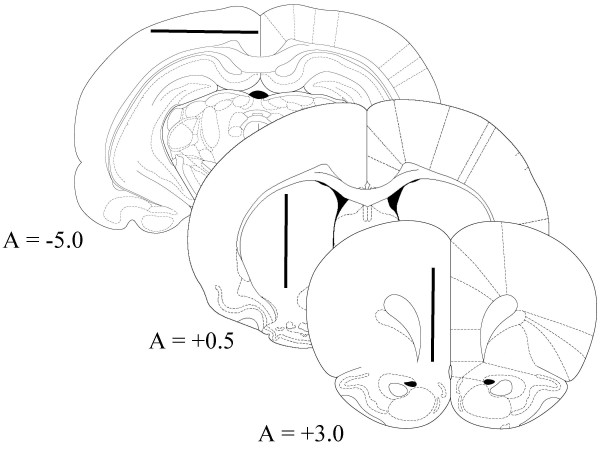
Schematic representations (redrawn from Paxinos and Watson's Atlas, 1997) of the cerebral areas implanted with dialysis probes.

**Figure 2:**
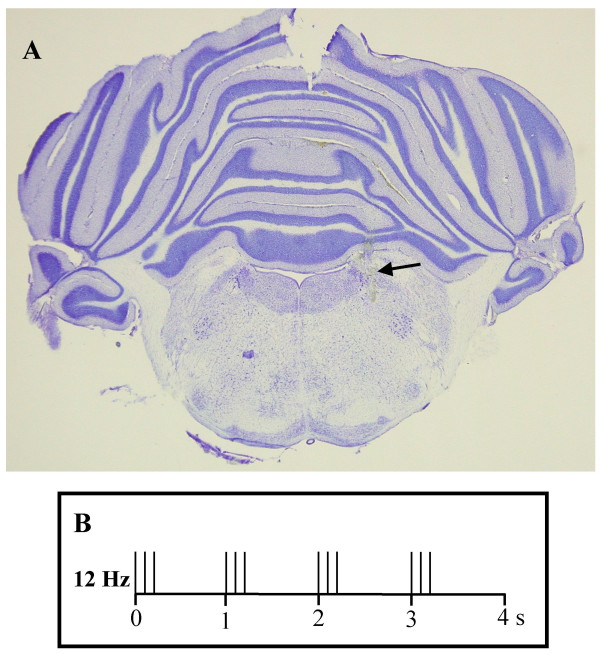
A: microphotograph showing the electrode localization into the locus coeruleus. B: schematic representation of locus coeruleus stimulation parameters; each vertical line represent a stimulus (700 μA, 250 μs), burst were delivered in 250 ms.

Positioning of the microdialysis probe is schematically depicted in fig. 1, while fig. 2 shows a microphotograph of electrode placement (A) and a schematic representation of the stimulation pattern used (B).

Mean extracellular basal levels of NA and DA, expressed as pg/sample (40 μl) injected on column were: in the mPFC (n = 25) 4.26 ± 0.49 and 4.34 ± 0.46, in the Occ (n = 26) 4.90 ± 0.65 and 4.22 ± 0.58, in the caudate nucleus (n = 6) 1.71 ± 0.16 and 27.35 ± 3.35 for NA and DA, respectively.

**Figure 3:**
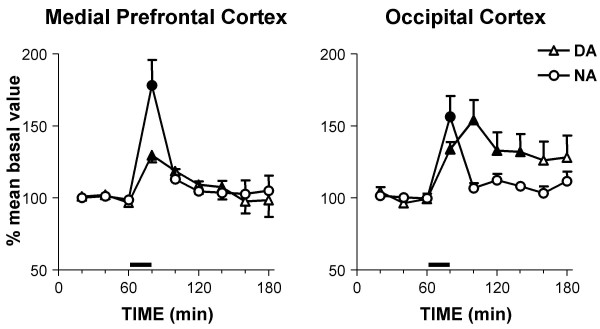
**Effect of locus coeruleus stimulation on extracellular NA and DA levels in the medial prefrontal and occipital cortex**. Stimuli were delivered in bursts, as described in Methods, for a duration of 20 min (horizontal solid lines). Data are means ± SE, expressed as percent of mean basal value. Filled symbols indicate p < 0.05 with respect to basal values (Dunnett test)

Fig 3 shows how LC stimulation increased extracellular NA by 78 and 56%, in the mPFC and Occ, respectively, the levels returning to baseline values within 20 min (ANOVA results: mPFC: F_(8,40) _= 16.103, P < 0.0001; Occ: F_(8,40) _= 11.349, P < 0.0001). Extracellular DA concentration in the mPFC increased by 29%, and returned to baseline within 20 min. In the Occ extracellular DA reached a maximal increase of about 54% at 20 min after the end of stimulation and remained elevated by about 30% throughout the experimental period (ANOVA results: mPFC: F_(8,40) _= 5.043, P = 0.0002; Occ: F_(8,40) _= 5.386, P = 0.0001).

**Figure 4:**
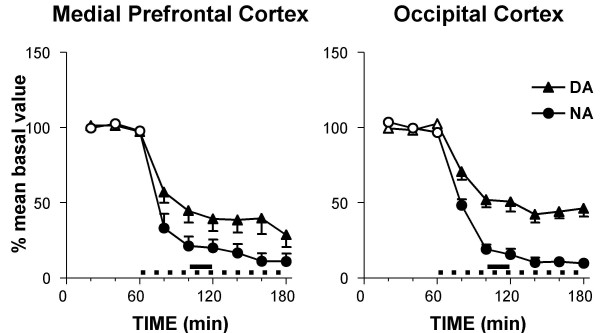
**Effect of locus coeruleus stimulation on extracellular NA and DA levels in the medial prefrontal and occipital cortex locally perfused with TTX**. TTX perfusion started after collection of basal samples, as indicated by the dashed line. Burst stimuli were delivered 40 min (two samples) after the beginning of TTX perfusion, for a duration 20 min (horizontal solid line). Data are means ± SE, expressed as percent of mean basal value. Filled symbols indicate p < 0.05 with respect to basal values (Dunnett test).

In order to ensure that extracellular DA represented a nerve activity-mediated exocytotic process, the mPFC and Occ were perfused with the sodium channel blocker tetrodotoxin (TTX,10 μM). TTX markedly decreased extracellular NA and DA (to about 10 and 30% of baseline, respectively) and, as shown in fig. 4, totally suppressed the increase in both catecholamines produced by electrical stimulation.

**Figure 5:**
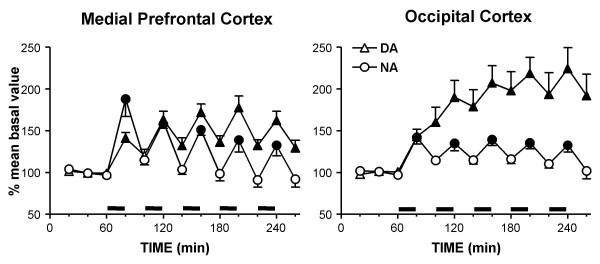
**Effect of repeated locus coeruleus stimulation on extracellular NA and DA levels in the medial prefrontal and occipital cortex**. Stimuli were delivered in bursts, as described in Methods (horizontal solid lines). Data are means ± SE, expressed as percent of mean basal value. Filled symbols indicate p < 0.05 with respect to basal values (Dunnett test).

A sequence of five stimulation trains was administered to a second group of rats, using the same burst pattern as for the single train stimulation, spaced out at 20 min interval (Fig 5). Each successive stimulation increased NA by approximately the same extent and duration as the first stimulation, NA returning to pre-stimulus level within 20 min after stimulation (ANOVA results: mPFC: F_(12,48) _= 12.329, P < 0.0001; Occ: F_(12,72) _= 13.086, P < 0.0001). In each interval sample NA levels differed significantly from those of the sample stimulated immediately previously, with the exception of the fifth pause in the mPFC and the second pause in the Occ (P < 0.05, Tukey-Kramer multiple comparison test).

On the other hand, following each stimulation DA remained elevated above the pre-stimulated level when the next stimulation was delivered, therefore the cumulative effect of the repeated stimulation was an increase in DA level by about 40 and 100% in the mPFC and Occ, respectively (ANOVA results: mPFC: F_(12,48) _= 17.402, P < 0.0001; Occ: F_(12,72) _= 18.714, P < 0.0001). In the mPFC, DA levels of the third, forth and fifth interval samples resulted significantly different from the sample stimulated previously, while in the Occ no significant difference was found between pauses and stimulations (P < 0.05, Tukey-Kramer multiple comparison test).

**Figure 6:**
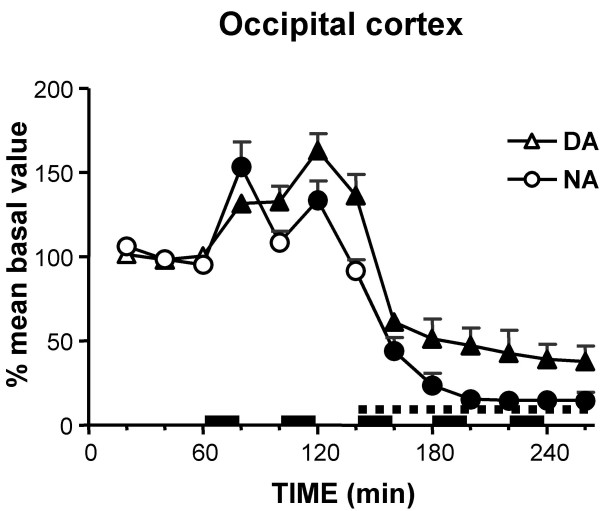
**Effect of TTX perfusion during repeated locus coeruleus stimulation on extracellular NA and DA levels in the occipital cortex**. Stimuli were delivered in bursts, as described in Methods (horizontal solid lines). TTX perfusion started with the third stimulation administration, as indicated by the dashed line. Data are means ± SE, expressed as percent of mean basal value. Filled symbols indicate p < 0.05 with respect to basal values (Dunnett test).

In a different group of rats, TTX was locally administered in the Occ after two stimulation periods, when DA level was elevated (Fig 6). This perfusion rapidly decreased extracellular NA and DA levels below basal concentrations, and the subsequent stimulations of LC totally failed to increase extracellular catecholamine levels (ANOVA results: NA: F(12,48) = 104.32, P < 0.0001; DA: F(12,48) = 40.806, P < 0.0001).

**Figure 7:**
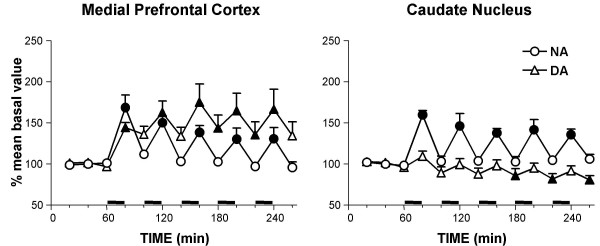
**Effect of repeated locus coeruleus stimulation on extracellular NA and DA levels in the medial prefrontal cortex and caudate nucleus. **Stimuli were delivered in bursts, as described in Methods (horizontal solid lines). Data are means ± SE, expressed as percent of mean basal value. Filled symbols indicate p < 0.05 with respect to basal values (Dunnett test).

To rule out the possibility that electrical stimulation might have produced the concomitant stimulation of the midbrain DA neurons by current spread or trans-synaptically, the effect of repeated burst stimulation of the LC on extracellular DA and NA in the caudate nucleus and the mPFC was compared in a group of rats implanted with a microdialysis probe in the two areas. As shown in Fig. 7, each successive stimulation increased extracellular NA and DA in the mPFC in the same way as in single probe implanted rats (ANOVA results: NA: F_(12,60) _= 14.309, P < 0.0001; DA: F_(12,60) _= 7.392, P < 0.0001). During each pause, NA levels were significantly different from the previously stimulated sample, while no difference was found between DA levels (P < 0.05, Tukey-Kramer multiple comparison test).

At variance to the cortex, in the caudate nucleus only extracellular NA levels were increased by stimulations, returning to baseline during each post-stimulus interval, while DA displayed an oscillatory tendency towards a decrease that reached significant values during the last 3 pauses (ANOVA results: NA: F_(12,60) _= 10.759, P < 0.0001; DA: F_(12,60) _= 5.000, P < 0.0001). As described for the cortex, during pauses NA levels were significantly different from the previously stimulated levels, with the exception of the fifth interval sample. Moreover, DA levels displayed a significant difference only between the first stimulated sample and the subsequent pause, although no significant difference was evidenced respect to baseline (P < 0.05, Tukey-Kramer multiple comparison test).

**Figure 8:**
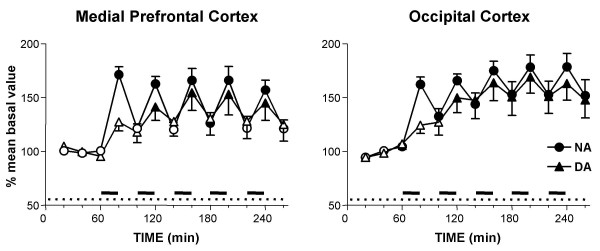
**Effect of repeated locus coeruleus stimulation on extracellular NA and DA levels in the medial prefrontal and occipital cortex locally perfused with DMI**. 100 μM DMI was administered through reverse dialysis for at least 2 h before stimulation, as represented by the dotted line. Stimuli were delivered in bursts, as described in Methods, for a duration 20 min (horizontal solid lines). Data are means ± SE, expressed as percent of mean basal value. Filled symbols indicate p < 0.05 with respect to basal values (Dunnett test).

A subset of experiments was performed during local perfusion of the NA transporter (NAT) blocker desipramine (DMI, 100 μM) into the mPFC and Occ. After at least 2 h of perfusion, extracellular NA and DA levels had increased stably, to 15.16 ± 1.93 and 8.27 ± 1.23 in the mPFC (n = 7), and to 19.60 ± 3,49 and 10.10 ± 3.53 pg/sample (n = 5) in the Occ, for NA and DA respectively. Multiple electrical stimulation of the LC further increased extracellular NA and DA levels, although in this condition not only DA but also NA failed to return to baseline during stimulation intervals, accumulating a significant increase of about 20% in the mPFC and 50% in the Occ (fig 8) (ANOVA results: mPFC, NA: F_(12,48) _= 20.743, P < 0.0001, DA: F_(12,48) _= 2.418, P = 0.0152; Occ, NA: F_(12,48) _= 21.852, P < 0.0001, DA: F_(12,48) _= 10.021, P < 0.0001). In the mPFC, each stimulated NA level was significantly different from the subsequent interval sample, while no significance was found in the Occ between stimulated and pause NA levels, as well as for DA levels in either the mPFC or Occ (P < 0.05, Tukey-Kramer multiple comparison test).

**Figure 9:**
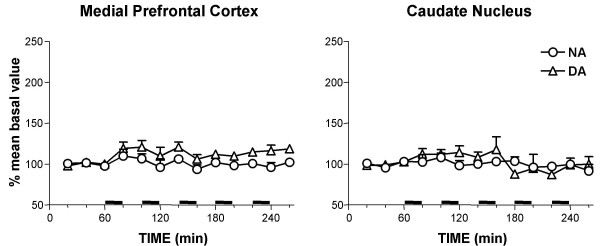
**Effect of stimulation outside the locus coeruleus on extracellular NA and DA levels in the medial prefrontal cortex and caudate nucleus**. Stimuli were delivered in bursts, as described in Methods (horizontal solid lines). Data are means ± SE, expressed as percent of mean basal value.

Fig 9 shows the results obtained in 3 rats whose data were discarded after histological examination, due to incorrect electrode positioning. In these animals neither extracellular NA nor DA were significantly increased by electrical stimulation, either in the mPFC (ANOVA results: NA: F_(12,24) _= 1.727, P > 0.05; DA: F_(12,24) _= 1.854, P < 0.05) or in the caudate nucleus (ANOVA results: NA: F_(12,24) _= 0.453, P > 0.05; DA: F_(12,24) _= 1.758, P > 0.05).

## Discussion

In this research study on anaesthetised animals we found that extracellular basal levels of NA and DA in the mPFC and Occ were analogous to those previously detected in our lab in freely moving rats. Due to the different probe geometry, which may have affected in vivo recovery of catecholamines, comparison of data collected in the mPFC (by means of vertical probes) with data obtained from Occ (by means of horizontal probes) is not appropriate. However, considering each area separately, it appears that while in the mPFC DA and NA concentrations were of a similar magnitude, consistent with the dense dopaminergic innervation of the mPFC, in the Occ DA resulted slightly lower than NA, but higher than one could expect from the scarce, if any, dopaminergic innervation, confirming our previous observations [8].

Electrical stimulation of the LC produced an increase in both NA and DA concentrations in the cerebral cortex of anaesthetized rats, superimposable to that previously demonstrated by our group in freely moving animals [12]. These data exclude the possibility that stress is involved in electrical stimulation induced catecholamine release.

Our results show that single train and repetitive stimulation of the LC caused a concomitant increase not only of extracellular NA but also of DA in both the mPFC and Occ.

Basal catecholamine levels and stimulation-induced elevations appear to be produced by a nerve-impulse mediated exocytotic process since the local perfusion of TTX dramatically reduced basal levels and totally suppressed the effect of LC stimulation in both cortices. This result exclude the possibility that the increase of extracellular DA originates from a passive leakage of cytosolic DA.

Furthermore, LC stimulation failed to increase DA in the caudate nucleus, which argues against the possibility that midbrain DA neurons might be stimulated by current spread or transynaptically.

NAT displays a high affinity for DA *in vitro*, and actively reuptakes it from extracellular space [17,18]. The coupling of NA and DA changes in the mPFC has been explained with the hypothesis that DA changes are secondary to the changes in NA competing with DA for NAT [19-21]. However, against this hypothesis is the finding that following phasic stimulation, DA remained elevated much longer than NA both in the mPFC and to a much longer extent in the OCC.

Moreover, electrical stimulation of the LC produced an increase in DA output when NA uptake had been previously blocked by a NAT inhibitor, both in the mPFC and Occ.

On the other hand, our results indicate that the time course of the release of the two catecholamines may be differentially influenced by the frequency and pattern of noradrenergic activity. Thus, while NA returned to the pre-stimulus level within 20 min after either single train or repeated phasic stimulation, DA elevation persisted for a much longer time. Indeed, repeated stimulation produced a persistent elevation of basal DA both in the mPFC and, to a greater extent, in the OCC. A similar elevation of inter-stimulation levels is also observed for NA during DMI perfusion, leading to the speculation that re-uptake mechanism *in vivo *is more active in clearing NA than DA, especially in the Occ. In fact, when NAT is blocked by DMI, NA curve approaches the DA one that, on the contrary, displays a similar profile in the absence or in the presence of DMI. A scarce efficacy of NAT in the clearance of DA from extracellular space in the Occ has been already proposed by Valentini et al. [22].

Another possible explanation for the finding that the duration of DA increase in both cortices exceeded that of NA, is that LC stimulation activates DA synthesis in noradrenergic neurons in excess to that can be converted to NA, due to increase in tyrosine hydroxylase activity following nerve cell stimulation [23], and consequent possible partial saturation of DA-β-hydroxylase. However, there is no report of this type of saturation, even though evidence exists for a stimulation-induced increase in tyrosine hydroxylase with no change in DA-β-hydroxylase activity in noradrenergic neurons [24-26].

A difficult problem is posed by the finding that DA elevation in the mPFC was of shorter duration than in the parietal cortex. It might be suggested that in the mPFC the DA transporter (DAT), other than NAT, participates in the removal of DA from extracellular space, so that DA removal from mPFC would be more efficient than in the OCC, where DAT is scarce [9,27]. The latter hypothesis is difficult to test, as the selective DAT inhibitor GBR 12909 has been demonstrated to be ineffective in raising extracellular DA levels in the mPFC [11,28] even during dopaminergic activity stimulation by means of haloperidol administration [11]. This observation has been explained with the NAT capacity to totally compensate for DAT exclusion, due to the high affinity of DA for NAT [17,18]. Anyway, this consideration does not exclude that in the mPFC DAT actively reuptakes DA into dopaminergic terminals, thus contributing to decrease extracellular DA levels. Hence, the different profiles of the curves might imply differences in clearance mechanisms for the two catecholamines. MAO A displays higher affinity for NA than for DA [29], and in the Occ DOPAC is about 10% that found in the mPFC [11], thus the possibility exists that in the Occ released DA is cleared prevalently by diffusion, instead of being metabolized to DOPAC.

By means of the double probe preparation, we demonstrated that the NA increase produced by LC electrical stimulation is elicited both in the mPFC and caudate nucleus, while DA increase is present in the mPFC but not in the caudate nucleus. Furthermore, when stimulating electrodes were placed outside the LC, no catecholamine increase was observed. Together, these data indicate that DA increase is not due to dopaminergic neuron activation by current spread. Moreover they exclude the possibility that the DA increase observed in the cerebral cortex is due to direct or indirect stimulation of dopaminergic neurons by NA; on the contrary, the DA decrease observed in the caudate nucleus during prolonged stimulation is consistent with an inhibitory action of NA on DA neurons, as already suggested [30,31]. Such an inhibitory action might contribute to explain why DA elevation in the mPFC was of shorter duration than in the occipital cortex: it might suggest that DA released from noradrenergic terminals was compensated in the mPFC by a reduced DA release from dopaminergic terminals. Indeed, in the caudate nucleus multiple stimulation evidenced a decrease in DA levels.

Finally, the finding that LC stimulation produced the same elevation of DA in areas with high or low dopaminergic innervation, argues against the possibility that a robust portion of extracellular DA, at least in the occipital cortex, might originate from the scarce dopaminergic afferences in these areas.

On the other hand, the co-transmitter hypothesis poses the problem of whether the release of the two catecholamines may be differentially controlled. Indeed, our results indicate that the time course of the release of the two catecholamines may be differentially influenced by the frequency and pattern of noradrenergic activity.

## Conclusion

In conclusion, results obtained in the present study provide further evidence that DA in the cerebral cortex satisfies the classic criteria for a candidate co-transmitter in NA neurons. Namely, DA is synthesized, stored, released and recaptured by NA neurons. Notably, the possibility that DA might be released from NA terminals may explain the presence of DA receptors in cortical areas where DA terminals are scarce or absent [3-7].

Our results also suggest that release of DA and NA from NA neurons might be differentially regulated depending on the pattern and frequency of neuronal activity. There is a great deal of evidence for a coupling between the release of DA and NA in the mPFC under different conditions including stress [14-16], antidepressants [32,33], psychostimulants [34,35], antipsychotic drugs [9,36,37], α_2_-adrenoceptor agonists and antagonists [8,10,11,38].

It would be of great interest to determine whether such coupling occurs in cortices outside the canonical mPFC, whether it represents the co-release of the two catecholamines from noradrenergic terminals, if the release of the two can be differentially controlled, and to understand the functional significance of such coupling.

## Methods

The experiments were performed on male Sprague-Dawley rats (Harlan Italy, S. Pietro al Natisone, Italy) weighing 270–300 g, housed in groups of 5 per cage prior to surgery and singly afterwards, at standard conditions of temperature and humidity and artificial light from 8 a.m. to 8 p.m.; food and water were available ad libitum. Experiments were approved by the local ethical committee and performed according to the guidelines for care and use of experimental animals of the UE (EEC Council 86/609; D.L. 27/01/1992, n. 116). Each animal, under Equithesin anesthesia (0.97 g pentobarbital, 2.1 g MgSO_4_, 4.25 g chloral hydrate, 42.8 ml propylene glycol, 11.5 ml 90% ethanol, distilled water up to 100 ml), was stereotaxically implanted, according to the coordinates of the atlas by Paxinos and Watson [39], with one vertical microdialysis probe in the mPFC (A +3.0, L ± 0.7, V -6.5, 4 mm active membrane length), or one transversal microdialysis probe in the OCC (A -5.0, V-1.8 from bregma, 4 mm active membrane length, corresponding to one hemisphere), or with two probes, a vertical one in the mPFC and a horizontal one in the homolateral OCC. In another group of animals, two vertical probes were inserted, one in the mPFC and one in the homolateral caudate nucleus (A +0.5, L ± 3.0, V -6.5 from bregma, 4 mm active membrane length). Dialysis membrane was AN 69-HF, Hospal-Dasco, Bologna, Italy; the *in vitro *recovery ranged from 14 to 20% for both DA and NA.

The day after probe implantation, animals were anaesthetized with chloral hydrate (400 mg/kg i.p.) and placed in a Kopf stereotaxic apparatus, where they remained throughout the experiment. Body temperature was maintained at 37°C with a heating pad, and anesthesia was maintained with a continuous infusion of chloral hydrate (400 mg/5 ml, i.p. infused at 0.28 ml/h). A standard bipolar stimulant electrode (Rhodes SNEX-100, Harvard Apparatus, Edenbridge, UK) was implanted in the locus coeruleus (A -3.1, V -8.4, L ± 1.3 from lambda, entering at 15° angle), homolateral to the vertical probes or to the dialyzing portion of the horizontal probe. Stimulant electrodes were connected to a Digitimer (Welwyn Garden City, UK) D4030 stimulus generator and the stimuli were delivered through a DC constant current generator-isolation unit Digitimer DS3. Stimuli were delivered in bursts (trains of 3 spikes regularly spaced in 250 msec, one train per sec) at an intensity of 700 μA, each spike lasting 250 μsec. This stimulation pattern was chosen, on the basis of previous results [12], as it elicited a consistent catecholamine increase with the least behavioural activation. Each rat received 1 stimulation train lasting 20 min or a sequence of 5 stimulation trains, spaced with 20 min pauses.

An artificial cerebrospinal fluid (147 mM NaCl, 4 mM KCl, 1.5 mM CaCl_2, _pH 6–6.5) was pumped through the dialysis probes at a constant rate of 2.2 μl/min via a CMA/100 microinjection pump (Carnegie Medicine, Stockholm, Sweden). Tetrodotoxin (TTX) and desipramine (DMI), purchased from Sigma-Aldrich (Milan, Italy), were dissolved in the artificial cerebrospinal fluid and administered by inverse dialysis at 10 and 100 μM concentration, respectively.

Samples were collected every 20 min, and NA and DA simultaneously analysed by high performance liquid chromatography (HPLC) apparatus with electrochemical detection, equipped with a 3.0 × 150 mm C18 (5 μ) Symmetry column (Waters) and an ESA Coulochem II detector. The mobile phase consisted of 80 mM Na_2_HPO_4_, 0.27 mM EDTA, 0.58 mM sodium octyl sulphate, 12% methanol, pH 2.8 with H_3_PO_4_, delivered by a model 307 Gilson pump at 0.4 ml/min; the Coulochem analytical cell first electrode was set at +200 mV, the second at -300 mV. In these conditions, the detection limits (signal to noise ratio 3:1) were 0.3 pg of NA and DA on column.

The average concentration of three stable samples (less than 15% variation) before treatment was considered as the control and was defined as 100%. Thus, values are expressed as percentages of controls + S.E.M.

On termination of experiments, rats were killed with an overdose of chloral hydrate (1.2 g/kg), the brains were removed and stored with 10% formalin in 0.2 M phosphate buffer until histologically verified; coronal sections (40 μm thick) were made with a Vibratome, oriented according to the atlas of Paxinos and Watson [39], stained with fast cresyl violet stain and the location of probes and electrodes was verified according to the above atlas. While no probe resulted outside the correct sampling area, electrodes were found outside the locus coeruleus in 8 out of 59 rats, the data from which were discarded, with the exception of data obtained in 3 of these rats that are shown separately.

Statistical significance was evaluated by analysis of variance (ANOVA) for repeated measures, followed by Dunnett multiple comparison test.

## Authors' contributions

PD designed and coordinated the study and participated in drafting the manuscript, GF and PS carried out the surgery and HPLC analysis, MF carried out the electrical stimulation and participated in the design of the study, GLG conceived the study and drafted the manuscript.
